# Exploring factors influencing childhood immunization status in East Africa using multilevel ordinal logistic regression analysis

**DOI:** 10.3389/fpubh.2024.1508303

**Published:** 2025-01-06

**Authors:** Aster Addisu Dires, Demeke Lakew Workie, Abay Kassa Teklie

**Affiliations:** Department of Statistics, Bahir Dar University, Bahir Dar, Ethiopia

**Keywords:** child immunization, ordinal logistic model, multilevel analysis, East Africa, status

## Abstract

**Introduction:**

Childhood vaccinations are crucial in safeguarding children from infectious diseases and are recognized as one of the most cost-effective public health interventions. However, children in East African countries face more than a fifteen-fold increased risk of death from vaccine-preventable diseases compared to those in high-income nations. This study aimed to identify the factors influencing childhood immunization status in East Africa.

**Methods:**

A sample of 22,734 children aged 12 to 23 months was included to assess immunization status, utilizing recent data from the Demographic and Health Survey conducted between 2015 and 2022 across ten East African countries. A Level-3 multilevel generalized odds model with a logit link function was employed for the analysis.

**Results:**

Among the 22,734 children in the sample, only 67.4% were fully immunized, 27.7% were partially immunized, and the remaining were not immunized at all. The null hypothesis of proportionality was rejected based on the Brant test. Consequently, various partial and non-proportional odds models were fitted, with the generalized odds model demonstrating the best fit compared to other ordinal regression models. The findings indicated that 43.14% of the variation in children’s immunization status was attributable to differences between countries, while 18.18% was due to variations between regions. Specific factors associated with immunization status revealed that mothers who attended antenatal care were 1.23 times more likely to fully immunize their children compared to those who did not, and those who received postnatal care were 1.13 times more likely to do so. Additionally, mothers who had antenatal and postnatal services were 1.07 and 1.08 times more likely, respectively, to fully or partially immunize their children compared to those who did not.

**Conclusion:**

The fitted generalized odds model indicated that several factors significantly associated with childhood immunization status included maternal age, number of antenatal and postnatal care visits, tetanus injections received by mothers, vitamin A intake, presence of health documentation, place of delivery, birth order, mother’s occupation, sex of the household head, distance to health facilities, maternal education, community maternal education, community wealth index, and community media exposure. Therefore, it is recommended that interventions focus on enhancing household wealth, educating mothers, and improving health systems.

## Introduction

Immunization serves as a straightforward, safe, and economical method to shield children from potentially dangerous diseases (1). To safeguard children against infectious diseases, vaccinations are administered either actively through vaccines or passively via immunoglobulin, which activates the immune system (2). Presently, basic childhood immunizations (such as BCG, pentavalent, polio, and measles) prevent an estimated 3.5 to 5 million deaths each year from diseases like tuberculosis, diphtheria, tetanus, pertussis, influenza, polio, and measles (3). However, in 2021, it was estimated that approximately 25 million children globally were either unvaccinated or inadequately vaccinated. Among these, around 18 million children did not receive any doses of the diphtheria-tetanus-pertussis (DTP) vaccine (4). This reduction in vaccination coverage has resulted in an increase in the number of unvaccinated children worldwide, rising from 13 million in 2019 to about 18 million in 2021, marking a nearly 40% increase. Furthermore, in 2022, there were still 20.5 million children lacking essential vaccines globally, which is 2.1 million more than in 2019 (5). The COVID−19 pandemic has also caused a significant decline in routine immunization rates, posing a serious risk to children’s health worldwide (6).

Africa has the highest under-five mortality rate globally, accounting for 40% of deaths in this age group, primarily due to vaccine-preventable diseases (VPDs) (7). Between 2019 and 2020, the number of zero-dose children remained stable at 0.3 million in the European Region but increased in the African Region from 7.1 million to 7.7 million (8). It is estimated that one in five African children does not receive all necessary vaccinations. As a result, over 30 million African children under five continue to suffer from VPDs each year, with more than 500,000 of these children dying annually (9). Nearly 44% of the 55,102 children aged 12 to 23 months enrolled in 25 surveys conducted in sub-Saharan Africa from 2013 to 2020 missed vaccination opportunities, indicating they were either unvaccinated or under-vaccinated (10).

The WHO immunization schedule indicates that East Africa has a low rate of childhood vaccination coverage, with only 69.21% of children aged 12 to 23 months receiving all recommended vaccinations. This figure varies significantly between countries. According to the UN statistics division, East Africa comprises 19 countries: Burundi, Comoros, Djibouti, Ethiopia, Eritrea, Kenya, Madagascar, Malawi, Mauritius, Mozambique, Reunion, Rwanda, Seychelles, Somalia, Somaliland, Tanzania, Uganda, Zambia, and Zimbabwe (11, 12) and in this region various factors influence a child’s vaccination status, including the child’s health, maternal healthcare, and socio-demographic characteristics (13).

However, when evaluating the reasons for complete or partial vaccination coverage in East Africa, critical factors such as the presence of unvaccinated children is often overlooked. While some studies have examined children’s vaccination status using two-level logistic regression models in various East African countries, none have employed three-level ordinal logistic regression analysis. The existing research has analyzed various factors affecting vaccination coverage, but aside from studies conducted in Ethiopia (14, 15) using two-level ordinal logistic regression and classical ordinal logistic regression models, the literature primarily relies on binary and multinomial logistic regression, categorizing vaccination status as fully vaccinated or not fully vaccinated. However, a child’s vaccination status is typically classified as fully vaccinated, partially vaccinated, or not vaccinated, considering the natural order of these categories. Despite previous studies exploring the prevalence and factors associated with complete basic childhood vaccination in East Africa (13, 16), there remains a gap in research specifically examining childhood vaccination status and related factors among children aged 12 to 23 months using pooled DHS data in this region.

Consequently, it is essential to investigate vaccination coverage and the factors influencing childhood vaccination status across East Africa to assess cross-national differences. This study aims to analyze recent pooled DHS data (2015–2022) from ten East African countries, focusing on factors affecting immunization status in children aged 12 to 23 months using three-level ordinal logistic regression analysis.

## Methods

### Data source and study design

This study utilized data from the latest Demographic and Health Surveys (DHS) conducted across ten East African countries. The selection of these countries was based on the availability of relevant variables and household cluster GPS coordinates (latitude and longitude). The DHS provides comprehensive country-level data from surveys conducted between 2015 and 2022 (Table 1). We chose DHS data because it is the most extensive resource for information regarding low- and middle-income countries. The analysis included a sample of 22,734 women with children aged 12 to 23 months. The data were extracted from the Kids Record (KR) files of the DHS, which is a nationwide survey carried out every five years in low- and middle-income nations.^1^ The DHS is representative of each country and emphasizes critical maternal and child health indicators, including the immunization status of children. A multistage sampling approach was implemented, beginning with the selection of clusters (enumeration areas, or EAs), followed by systematic sampling of households within these chosen EAs.

**Table 1:** Description of the sample size and the respective recent DHSs.

Country	Survey year	Total number of women’s having children aged 12–23 months old	
		Un-weighted sample	Weighted sample
Burundi	2016–17	2,585	2,669
Ethiopia	2016	1870	1943
Kenya	2022	3,534	3,158
Madagascar	2021	2,304	2,296
Malawi	2015–16	3,192	3,177
Rwanda	2019–20	1,558	1,617
Tanzania	2022	2088	2,130
Uganda	2016	2,787	2,718
Zambia	2018	1899	1861
Zimbabwe	2015	1,093	1,165
Total		22,910	22,734

### Variables of the study

#### Response variable

In this study, the response variable was defined as “children’s immunization status,” which is classified into three categories: fully immunized, partially (incompletely) immunized, and not immunized, in accordance with the national vaccination and Expanded Program on Immunization (EPI) schedule (17). According to the World Health Organization (WHO) guidelines, a child aged 12 to 23 months is considered not immunized if they have not received any of the eight required vaccines (one dose of BCG, at least three doses of the pentavalent vaccine, three doses of oral polio vaccine (OPV), and one dose of the measles vaccine), and is coded as “0.” A child is classified as partially (incompletely) immunized if they missed at least one of the eight vaccines, receiving a code of “1.” Conversely, a child is deemed fully immunized if they have received all eight vaccines, which is coded as “2” (13, 15, 18–22). Information on vaccination status for children comes from the DHS Kids Record (KR) files, which are designed to capture detailed health indicators, including immunization status. The vaccination status is categorized based on whether children received all required vaccines as per the national Expanded Program on Immunization (EPI) guidelines. The analysis considers the availability of health records as an independent variable, which is crucial since having proper health documentation can significantly influence immunization rates. This variable reflects the accessibility and utilization of healthcare services, underscoring its importance in understanding the broader context of vaccination coverage.

#### Independent variables

The selection of independent variables associated with children’s immunization status was informed by existing knowledge and previously published literature (7, 10, 14, 15, 22–25). The variables age of mother, marital status, mother education level, father education level, occupation of mother, occupation of father, sex of household head, access of mass media, number of children, birth order, birth interval, weight at birth, sex of child, presence of vaccination document, place of delivery, antenatal follow up, postnatal follow up, distance to health center, tetanus injection before birth, talking vitamin A_1_ were potential lower level variables, while community mother education, community wealth index, community media exposure, and residence were the potential higher level (level-two) variables.

### Statistical analysis

Data analysis was conducted using R version 4.3.3, where categorical variables were summarized by frequencies and percentages. To address the prevalent missing values in the DHS data, a multiple imputation technique was employed, restoring natural variability and accounting for uncertainty in statistical inference (26). Given the ordinal nature of the outcome variable, children’s immunization status (not immunized, partially, and fully immunized), a three-level ordinal logistic regression model was utilized. This hierarchical model recognizes that children are nested within regions or provinces, which are further nested within countries.

**Figure 1:**
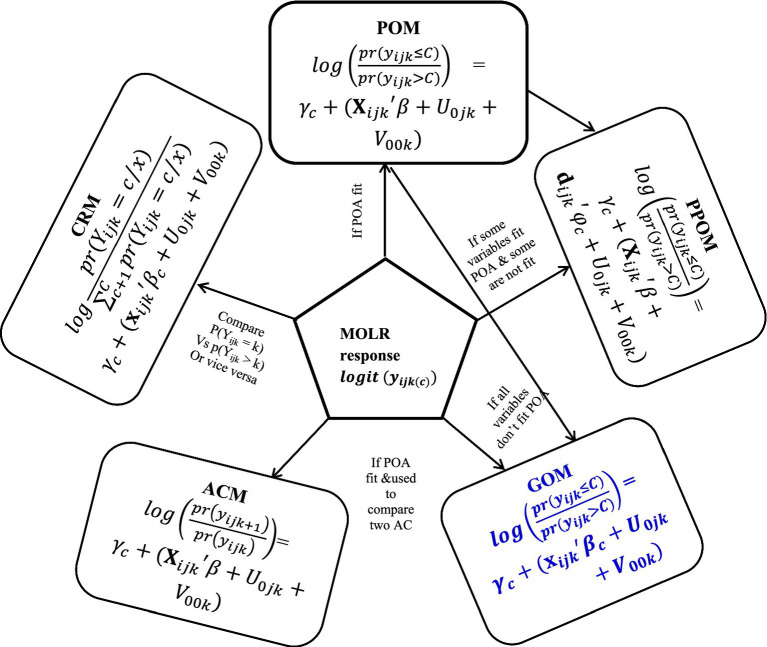
Different types of three-level ordinal logistic regression models.

The proportional odds (PO) assumption underlies this model; if satisfied, it allows for consistent effects of independent variables across outcome categories (27). If not, alternative models such as the Partial Proportional Odds Model (PPOM) (28), Generalized Ordered Logit Model (GOM) (29), Continuation Ratio Model (CRM) (30), and Adjacent-Categories Logit Model (ACM) (31) can be applied (Figure 1).

The PPOM accommodates variables that meet the PO assumption while allowing flexibility for those that do not. The GOM extends the ordinal logistic regression by relaxing the proportionality constraint, enabling varied effects of explanatory variables. The CRM allows for distinct intercepts and coefficients for each response category comparison, while the ACM focuses on estimating odds between adjacent categories of the ordinal response. Overall, the use of a three-level ordinal logistic regression model is appropriate for analyzing children’s immunization status, considering the hierarchical data structure and the necessity to address the proportional odds assumption.

### Model building, parameter estimation, and test of model adequacy

Model building can adopt either a top-down or bottom-up approach, with the latter being more prevalent due to its efficiency. The top-down method starts with a complex model, risking long computation times and convergence issues, while the bottom-up strategy begins with a simple intercept-only model and iteratively adds parameters, testing their significance after each inclusion (32, 33) and variables with *p*-value of 0.25 or much more will exclude from the final covariate analysis (34). To assess the variation in children’s immunization status across clusters, we employed the Likelihood Ratio (LR) test, Intra-class Correlation Coefficient (ICC), and Proportional Change in Variance (PCV). The ICC quantifies heterogeneity in immunization status between clusters, indicating the proportion of total variation attributable to cluster differences and the PCV measures the proportion of variance explained by predictors at each model level (35). Parameter estimation in this context involves maximizing the log-likelihood function using Full Maximum Likelihood (FML) or Restricted Maximum Likelihood (RML). RML is generally preferred as it reduces bias by accounting for fixed effects (36). Testing model fit includes assessing the significance of fixed and random effects, with the Wald ratio employed for hypothesis testing (32). Finally, model selection is guided by Akaike’s Information Criterion (AIC) and log-likelihood statistics, where the model with the lowest AIC and highest -2LL is considered the best fit for the data.

## Results

### Immunization status in East Africa

**Figure 2:**
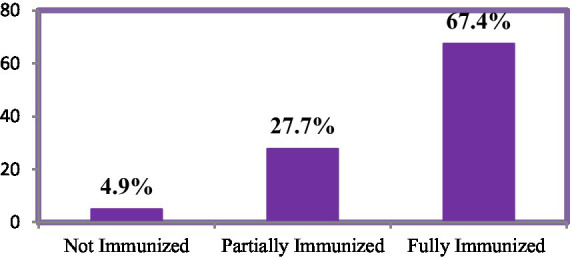
Children of age 12–23 months vaccination status in East Africa.

**Figure 3:**
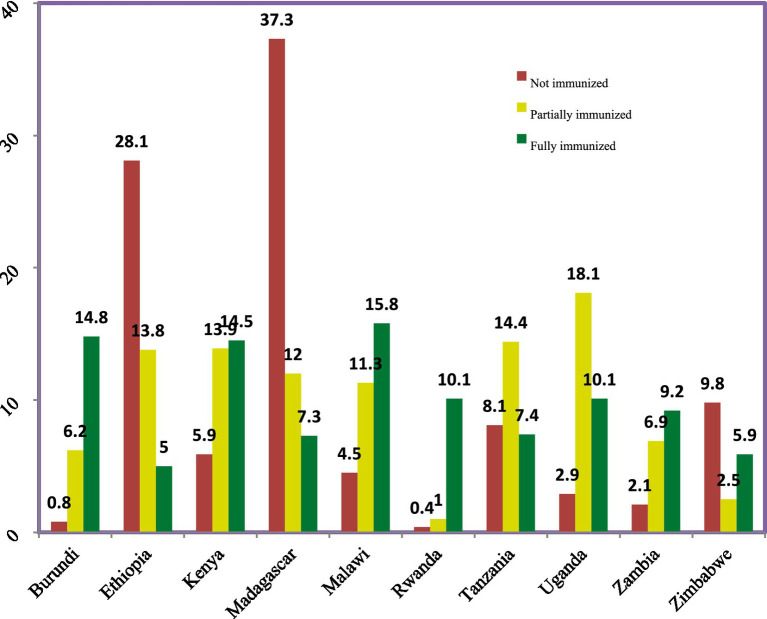
The prevalence of immunization status of children among East African countries.

In East Africa, the overall immunization coverage stands at 67.4%, with 27.7% of children being partially immunized and 4.9% not immunized at all. Among the 15,323 fully immunized children, the highest coverage is observed in Malawi at 15.8%, followed closely by Burundi at 14.8% and Kenya at 14.5%. Conversely, the rates of non-immunized children are particularly concerning in Madagascar, where 37.3% of children lack vaccinations, followed by Ethiopia at 28.1% and Zimbabwe at 9.8% (Figures 2, 3).

### Economic and socio-demographic characteristics of respondents and children

A total of 22,734 weighted women with children aged 12–23 months were included in the study, revealing that the majority of respondents, specifically 10,473 women, were in the 25–34 age group. Regarding parental educational status, approximately 11,419 mothers and 10,779 fathers had only primary education, and this group exhibited the highest proportion of children who were either not vaccinated or partially vaccinated. In contrast, parents with secondary education demonstrated significantly higher rates of full immunization for their children, with 74.1% of mothers and 71.4% of fathers achieving this status. Additionally, nearly 70% of the communities surveyed were classified as having a poor wealth index, and over 80% of the respondents lived in rural areas. These socio-demographic factors highlight the relationship between parental education and child vaccination rates, emphasizing the need for targeted interventions to improve immunization coverage, particularly among less educated populations (Table 2).

**Table 2:** Frequency distribution of covariates for different categories of immunization status.

Variables	Categories (codes)	Immunization Status		
		Not-immunized	Partially- immunized	Fully-immunized
Age of mother	15–24 (0)	386 (5.1)	2,181 (28.6)	5,062 (66.3)
	25–34 (1)	499 (4.8)	2,795 (26.7)	7,179 (28.4)
	35–49 (2)	233 (5)	1,315 (28.4)	3,084 (66.6)
Mother educational level	No education (1)	518 (11.3)	1,581 (34.5)	2,489 (54.3)
	Primary (2)	418 (3.7)	3,165 (27.7)	7,836 (68.6)
	Secondary (3)	161 (2.9)	1,282 (23)	4,127 (74.1)
	Higher (4)	22 (1.9)	262 (22.6)	873 (75.5)
Father educational level	No education (1)	415 (10.5)	1,298 (32.8)	2,244 (56.7)
	Primary (2)	429 (4)	3,021 (28)	7,329 (68)
	Secondary (3)	232 (3.7)	1,541 (24.9)	4,423 (71.4)
	Higher (4)	42 (2.3)	430 (23.9)	1,330 (73.8)
Marital status	Married (1)	832 (5.3)	4,213 (26.9)	10,604 (67.8)
	Not married (2)	286 (4)	2078 (29.4)	4,721 (66.6)
Occupation of father	Yes (1)	998 (4.7)	5,766 (27.4)	14,282 (67.9)
	No (2)	120 (7.1)	525 (30.1)	1,043 (61.)
Occupation of mother	Employed (1)	642 (4.1)	4,218 (26.7)	10,948 (69.3)
	House wife (2)	476 (6.9)	2073 (29.9)	4,377 (63.2)
Sex of household	Male (1)	871 (4.9)	4,814 (27.2)	12,005 (67.9)
	Female (2)	247 (4.9)	1,477 (29.3)	3,320 (65.8)
Household wealth index	Poor (1)	6,541 (63.7)	697 (6.8)	3,034 (29.5)
	Middle (2)	3,041 (68.7)	192 (4.3)	1,192 (26.9)
	Rich (3)	5,743 (71.5)	230 (2.8)	2065 (25.7)
Access to media exposure	Yes (1)	7,997 (71.2)	342 (3)	2,894 (25.8)
	No (2)	7,329 (63.8)	776 (6.7)	3,396 (29.5)
Number of living children	1–2 (0)	7,284 (70.2)	399 (3.8)	2,686 (25.9)
	3–4 (1)	4,776 (67.8)	323 (4.6)	1941 (27.6)
	5+ (2)	3,264 (61.3)	397 (7.5)	1,664 (31.2)
Birth order of child	First order (1)	203 (3.8)	1,346 (25.1)	3,820 (71.1)
	2–3 (2)	361 (4.3)	2,216 (26.1)	5,917 (69.7)
	4 and above (3)	555 (6.3)	2,728 (30.7)	5,589 (63)
Birth interval of child	<=23 months (1)	226 (8.2)	927 (33.5)	1,615 (58.3)
	24-47 months (2)	596 (4.8)	3,399 (27.8)	8,218 (67.3)
	> = 48 months (3)	296 (4)	1964 (25.3)	5,494 (70.7)
Weight of child	Small (1)	370 (5.5)	1900 (27.9)	4,531 (66.6)
	Average (2)	487 (4)	3,265 (27)	8,356 (69)
	Large (3)	262 (6.8)	1,126 (29.4)	2,439 (63.7)
Sex of child	Male (1)	566 (5)	3,178 (27.9)	7,654 (62.1)
	Female (2)	552 (4.9)	3,113 (27.4)	7,671 (67.7)
Health documentation	Yes (1)	117 (0.6)	3,844 (21.3)	14,102 (78.1)
	No (2)	1,002 (21.5)	2,446 (52.4)	1,223 (26.2)
Place of delivery	Home (1)	782 (15.6)	1937 (38.4)	2,308 (46)
	Health facility (2)	336 (1.9)	4,354 (24.6)	13,018 (73.5)
ANC	Yes (1)	663 (3.1)	5,736 (26.9)	14,910 (70)
	No (2)	455 (31.9)	555 (38.9)	415 (29.2)
PNC	Yes (1)	201 (3.0)	1788 (25)	5,164 (72)
	No (2)	918 (5.9)	4,502 (28.8)	10,161 (65.3)
Distance to health Facility	Big problem (1)	592 (6.7)	2,644 (29.8)	5,620 (63.5)
	Not Big problem (2)	526 (3.8)	3,647 (26.3)	9,705 (69.9)
Tetanus injection	Not received (1)	591 (11.5)	1,406 (27.4)	3,131 (61.1)
	1–2 (2)	355 (2.6)	3,619 (26.7)	9,598 (70.7)
	3 and above (3)	172 (4.3)	1,265 (31.3)	2,597 (64.4)
Vitamin A_1_	Yes (1)	284 (1.7)	3,965 (23.1)	12,908 (75.2)
	No (2)	835 (15)	2,325 (41.7)	2,417 (43.3)
Residence	Urban (1)	121 (2.5)	1,371 (28.3)	3,347 (69.2)
	Rural (2)	998 (5.6)	4,919 (27.5)	11,978 (66.9)
Community mother education	Low (1)	518 (11.3)	1,581 (34.5)	2,489 (54.3)
	High (2)	600 (3.3)	4,710 (26)	12,836 (70.7)
Community wealth index	Low (1)	889 (6.0)	4,226 (28.8)	9,582 (65.2)
	High (2)	229 (2.8)	2065 (25.7)	5,743 (71.5)
Community media exposure	Low (1)	809 (6.1)	3,542 (26.9)	8,829 (67)
	High (2)	310 (3.2)	2,798 (28.8)	6,496 (68)

### Model comparison for the three-level model

Due to the violation of the parallel line regression assumption (Brant test) ( $x^{2}:$ 978.23, *p*-value = 0.000), the PO model was excluded. Subsequently, various other models were applied to the data. Among the models considered, the GOM model exhibited the best fit based on AIC, BIC, and -2LL values (Table 3).

**Table 3:** Model comparison on different three level OLR models.

Type	AIC	BIC	-2LL	*p*-value
PPOM	8,69.625	1,116.144	-1,267.5	<0.000
**GOM**	**8,865.602**	**1,115.817**	**-1,062.3**	**<0.000**
ACM	8,865.835	1,115.833	-1,475.2	<0.000
CRM	8,869.145	1,116.163	-1,478.8	<0.000

The bold values indicates that the best fit model based on AIC, BIC, and -2LL values.

### Factors associated with experiencing children immunization status

The GOM results for the fixed effects in Table 4 present two comparisons: the first contrasts fully immunized children with those who are partially or not immunized, while the second compares fully or partially immunized children to those who are not immunized, from left to right in the table. In the univariate analysis except sex of child all other variables were significantly associated with child immunization status. But based on these results at a 5% level of significance, factors significantly associated with childhood immunization status include the mother’s age, antenatal care (ANC) visits, postnatal care (PNC) visits, the number of tetanus injections received by mothers, vitamin A supplementation, presence of health documentation, number of children, place of delivery, mother’s occupation, sex of the household head, distance to the health facility, mother’s education, residence, community-level maternal education, community-level wealth index, and community-level media exposure.

**Table 4:** Maximum likelihood estimates of fixed effects.

		Fully vs partially or not immunized		Fully or partially vs not immunized	
Predictors	Categories	AOR (95%CI)	*p*-value	AOR (95%CI)	*p*-value
Intercept	-	0.04 (0.0126, 0.0674)	<0.001***	0.06 (0.0.04, 0.08)	<0.001***
Mother’s age	15–24	1		1	
	25–34	1.14 (1.0773, 1.2027)	0.0017**	1.12 (0.9671, 1.2729)	0.126
	35–49	1.24 (1.1988, 1.2812)	<0.001***	1.25 (1.2088, 1.2912)	<0.001***
ANC	No	1		1	
	Yes	1.23 (1.1300, 1.3300)	<0.001***	1.07 (1.0092, 1.1307)	0.0062**
PNC	No	1		1	
	Yes	1.13 (1.1084, 1.1516)	<0.001***	1.08 (1.0584, 1.1016)	0.0011**
TT	Not received	1		1	
	1–2	1.02 (0.9808, 1.0592)	0.867	1.05 (1.0050, 1.0951)	0.0166*
	3 and above	1.36 (1.2953, 1.4247)	<0.001***	1.21 (1.1061, 1.3139)	<0.001***
Vitamin A_1_	No	1		1	
	Yes	1.06 (1.0169, 1.1031)	0.019*	1.23 (1.0281, 1.4319)	0.0044
Health documentation	No	1		1	
	Yes	1.11 (1.0473, 1.1727)	0.0035**	1.38 (1.2604, 1.4996)	<0.001***
Place of delivery	Home	1		1	
	Health facility	1.12 (1.0396, 1.2004)	<0.001***	1.34 (1.2460, 1.4341)	<0.001***
Distance to health facility	Not big problem	1		1	
	Big problem	0.86 (0.7757, 0.9443)	0.048*	0.88 (0.7938, 0.9662)	0.0086**
Sex of house hold head	Male	1		1	
	Female	0.51 (0.4081, 0.6119)	<0.001***	0.41 (0.3081, 0.5119)	<0.001***
Number of Child	1–2	1		1	
	3–4	0.44 (0.37,0.51)	<0.001***	0.53 (0.33, 0.73)	<0.001***
	5+	0.22 (0.14, 1.30)	0.3306	0.18 (0.11,1.20)	0.2511
Occupation of Mother	House wife	1		1	
	Employed	0.80 (0.7216, 0.8784)	<0.001***	0.73 (0.6321, 0.8279)	<0.001***
Mother education	No education	1		1	
	Primary	1.13 (0.9281, 1.3319)	0.261	1.23 (0.99, 1.47)	0.0266
	Secondary	1.30 (1.0981, 1.5019)	<0.001***	1.11 (1.0865, 1.1335)	<0.001***
	Higher	1.87 (1.7504, 1.9896)	<0.001***	1.25 (1.0716, 1.4284)	<0.001***
Residence	Urban	1		1	
	Rural	0.55 (0.51, 0.59)	<0.001***	0.48 (0.42, 0.54)	<0.001***
Community wealth Index	Low	1		1	
	High	1.33 (1.2849, 1.3751)	<0.001***	1.34 (1.2028, 1.4772)	<0.001***
Community media exposure	Low	1		1	
	High	1.18 (1.1369, 1.2231)	<0.001***	1.22 (1.0906, 1.3494)	<0.001***
Community mother education	Low	1		1	
	High	1.40 (1.3392, 1.4608)	<0.001***	1.37 (1.3288, 1.4112)	<0.001***

*** (*p* < 0.001), ** (*p* < 0.01), * (*p* < 0.05). CI, Confidence interval; AOR, Adjusted Odds Ratio, 1 = estimate value for reference group, and ---have no categories (for continuous variable).

Table 4 indicates that, controlling for other factors, children whose mothers are aged 25–34 and 35–49 years have 1.14 [OR = 1.14, CI: 1.08–1.20] and 1.24 [OR = 1.24, CI: 1.20–1.28] times higher odds of being fully immunized compared to those whose mothers are aged 15–24. Additionally, the odds of children aged 12–23 months being fully immunized are 1.23 [OR = 1.23, *p* = 0.001] times higher if their mothers had antenatal care. Children whose mothers received postnatal care have odds of being fully immunized that are 1.13 [OR = 1.13, *p* = 0.001] times higher. For tetanus injections, children born to mothers with 3+ injections are 1.36 [OR = 1.36, CI: 1.30–1.42] times more likely to be fully immunized compared to those with no injections. The odds increase to 1.06 [OR = 1.06, CI: 1.02–1.10] for children whose mothers received vitamin A. The model shows that children with health documentation have 1.11 [OR = 1.11, *p* = 0.0035] times higher odds of being fully immunized. Children born in health facilities are also 1.12 [OR = 1.12, CI: 1.04–1.20] times more likely to be fully immunized. Educational attainment also plays a role; children of mothers with secondary or higher education have 1.30 [OR = 1.30, CI: 1.10–1.50] and 1.87 [OR = 1.87, CI: 1.75–1.99] times higher odds of full immunization than those of non-educated mothers.

Distance to health facilities negatively impacts immunization; children whose mothers perceive distance as a major problem have lower odds of being fully immunized [OR = 0.86, CI: 0.78–0.94]. Female-headed households show a reduced likelihood of full immunization compared to male-headed households [0.51 times]. Children from larger families (3–4 or 5+ children) are significantly less likely to be fully immunized compared to those from families with 1–2 children [OR = 0.44 and 0.22, respectively]. Employed mothers are 0.8 [OR = 0.8, CI: 0.72–0.88] times less likely to fully vaccinate their children compared to housewives. Rural children are 0.55 [OR = 0.55, CI: 0.51–0.59] times less likely to be fully immunized than those in urban areas. Communities with higher wealth indices and media exposure correlate with higher immunization rates [OR = 1.33 and 1.18, respectively]. Finally, children in communities with high maternal education levels are 1.40 [OR = 1.40, CI: 1.34–1.46] times more likely to be fully immunized compared to those in communities with low maternal education.

The random component output variance at the country and region level decreased from 3.67 and 1.55 to 2.8 and 1.3, respectively. This decrement suggests that the inclusion of the predictors explains some of the variation at both the country and regional levels compared to the null model (Table 5). Also, it reflects that the proportion of explained variance (PCV) for the country level in relation to child immunization status, by considering both level − 1 and level-2 predictors, were 23.7%. This means that 23.7% of the variability in child immunization status at the country level could be accounted for these predictors. Similarly, the PCV for the region level is 0.161, indicating that 16.1% of the variability at the level-2 (region) is explained by the combination of level-1 and level-2 predictors.

**Table 5:** Maximum likelihood estimates of random effect.

Estimate	Subject	Estimate	Std. Error	AOR (95% CI)
Intercept	Country	1.03	0.021	2.8 (2.69,2.92) ***
Intercept	Region (country)	0.26	0.011	1.3 (1.27,1.33) ***
PCV country	0.237	-	-	-
PCV region	0.161	-	-	-

PCV, proportional change in variance, and *** (*p* < 0.001), Std. Error, standard error; CI, Confidence interval; OR, Odds Ratio.

## Discussion

The objective of this study was to investigate the factors that influence childhood immunization status in ten East African countries, comprising a total of 132 regions based on recent DHS data.

In this study, a single outcome measure of “immunization status of children” was computed based on the World Health Organization (WHO) guidelines of the eight recommended vaccine types, and recoded into ordinal outcome. Various models, including PPOM, GOM, ACM, and CRM, were applied to the data, and a comparison of these models was conducted. According to the AIC and BIC criteria, the GOM model demonstrated the best fit. Consequently, the GOM model was selected to identify significant determinants of a child’s immunization status. Among the five models considered in this study, the fourth (Random coefficient) model yielded the best parameter estimates. Therefore, the significant predictors in the selected model were presented and interpreted at a 5% significance level.

The study revealed an overall full vaccination coverage of 67.4% (15,322.716 children) and the highest proportions of fully immunized children were observed in Malawi (15.8%), followed by Burundi (14.8%) and Kenya (14.5%). Conversely, Madagascar (37.3%), Ethiopia (28.1%), and Zimbabwe (9.8%) had the highest rates of non-immunized children. These findings align with existing literature on childhood vaccination in East Africa but indicate lower rates than those reported in Pakistan, possibly due to differences in healthcare infrastructure, access to services, and survey methodologies (13, 37). Key predictors of immunization status identified through the GOM model included maternal age, antenatal care (ANC) visits, postnatal care (PNC) visits, tetanus injections received, vitamin A1 supplementation, health documentation, place of delivery, maternal occupation, household head’s sex, number of children, distance to health facilities, maternal education, community wealth index, and media exposure. Children with mothers aged 15–24 were less likely to be fully immunized compared to those with older mothers, supporting findings from similar studies in the region (13, 14, 22, 38). Antenatal care visits were a strong predictor of childhood immunization, as they provide critical education about vaccination benefits and schedules (13, 15, 19, 20, 39–41). Similarly, children whose mothers attended postnatal check-ups had higher odds of being fully or partially immunized, reinforcing the importance of continuous healthcare engagement (15). The study also found that mothers who received tetanus injections during pregnancy were more likely to ensure their children were fully immunized. This trust in healthcare providers, developed through positive vaccination experiences, significantly influences childhood immunization rates (23, 42). Vitamin A1 supplementation during pregnancy was associated with higher immunization rates, likely due to increased interaction with healthcare services (22). Presence of health documentation was another significant factor, as mothers with vaccination records were more likely to have fully vaccinated children. This suggests that documentation reinforces accountability and facilitates adherence to immunization schedules (22, 23). Delivery location significantly impacted immunization status, with children born in healthcare facilities having higher immunization rates than those born at home. This is attributed to immediate vaccination opportunities provided during facility births, such as the BCG vaccine (10, 13–15, 19, 43–45). Distance to health facilities posed a considerable barrier to immunization, with mothers reporting distance as a significant issue having lower odds of fully immunizing their children. This aligns with findings from multiple studies indicating that access to healthcare resources is crucial for vaccination uptake (7). The analysis also indicated that children from female-headed households were less likely to be fully immunized compared to those from male-headed households. This may be due to additional time constraints faced by female heads of households, impacting their ability to prioritize immunization appointments (15, 40). Families with a higher number of children aged 12–23 months showed lower immunization rates, as larger family size negatively correlates with vaccination status. This is consistent with literature suggesting that resource allocation and parental engagement diminish with increased child numbers (15).

Maternal employment status also influenced immunization rates, with employed mothers less likely to fully vaccinate their children. This could be due to time constraints that make it challenging to attend vaccination appointments. Educational attainment among mothers was positively associated with children’s immunization status. Children of mothers with secondary or higher education were more likely to be fully vaccinated, highlighting the role of education in accessing reliable health information and resources (10, 13–15, 22–24, 40, 43–45). Rural residency was linked to lower vaccination rates compared to urban areas, likely due to socioeconomic factors, including lower parental education and limited access to healthcare facilities (14, 15, 19, 22, 23, 39, 40, 45). Community wealth index emerged as a significant predictor of immunization status, with children from wealthier communities having higher odds of being fully vaccinated. This suggests that socioeconomic status directly influences access to healthcare services (13, 23). Finally, community-level media exposure and maternal education were significantly affected vaccination rates. Children in a community with higher media exposure and maternal education were more likely to be fully immunized, as media can play a crucial role in educating and motivating parents about the importance of vaccinations (23).

## Conclusion

This study highlights the critical issue of immunization coverage among children aged 12–23 months in East Africa. The findings indicate significant disparities in immunization rates, particularly in Madagascar, Ethiopia, and Zimbabwe, where non-immunization rates are notably high. A three-level ordinal logistic regression analysis identified key predictors of immunization status, including maternal age, antenatal and postnatal care visits, tetanus injections, vitamin A supplementation, and various community factors. These results underscore the ongoing challenges in achieving comprehensive immunization coverage in the region. Authors recommend that enhancing maternal education, improving healthcare access, engaging communities, and addressing socioeconomic barriers to boost childhood vaccination rates across the region. Programs that elevate maternal education led to better-informed mothers who seek vaccinations. Strengthening access to antenatal and postnatal care is essential, as is promoting community awareness of vaccination. Finally, improving household wealth and reducing poverty are vital, as economic stability correlates with better health outcomes and higher vaccination rates.

## Data Availability

Publicly available datasets were analyzed in this study. This data can be found at: https://www.dhsprogram.com/Data.

## Footnotes

^1^ https://www.dhsprogram.com/Data
